# The Universal Statistical Distributions of the Affinity, Equilibrium Constants, Kinetics and Specificity in Biomolecular Recognition

**DOI:** 10.1371/journal.pcbi.1004212

**Published:** 2015-04-17

**Authors:** Xiliang Zheng, Jin Wang

**Affiliations:** 1 State Key Laboratory of Electroanalytical Chemistry, Changchun Institute of Applied Chemistry, Chinese Academy of Sciences, ChangChun, Jilin, P.R. China; 2 Department of Chemistry and Physics, State University of New York at Stony Brook, Stony Brook, New York, United States of America; Fudan University, CHINA

## Abstract

We uncovered the universal statistical laws for the biomolecular recognition/binding process. We quantified the statistical energy landscapes for binding, from which we can characterize the distributions of the binding free energy (affinity), the equilibrium constants, the kinetics and the specificity by exploring the different ligands binding with a particular receptor. The results of the analytical studies are confirmed by the microscopic flexible docking simulations. The distribution of binding affinity is Gaussian around the mean and becomes exponential near the tail. The equilibrium constants of the binding follow a log-normal distribution around the mean and a power law distribution in the tail. The intrinsic specificity for biomolecular recognition measures the degree of discrimination of native versus non-native binding and the optimization of which becomes the maximization of the ratio of the free energy gap between the native state and the average of non-native states versus the roughness measured by the variance of the free energy landscape around its mean. The intrinsic specificity obeys a Gaussian distribution near the mean and an exponential distribution near the tail. Furthermore, the kinetics of binding follows a log-normal distribution near the mean and a power law distribution at the tail. Our study provides new insights into the statistical nature of thermodynamics, kinetics and function from different ligands binding with a specific receptor or equivalently specific ligand binding with different receptors. The elucidation of distributions of the kinetics and free energy has guiding roles in studying biomolecular recognition and function through small-molecule evolution and chemical genetics.

## Author Summary

Uncovering the principles and underlying mechanisms of biomolecular recognition and molecular binding process is crucial for understanding the function and evolution, yet challenging. We meet the challenge by quantifying the statistical natures of the relevant physical variables of biomolecular recognition using the analytical model combined with microscopic flexible docking simulation methods. We uncovered the universal statistical laws obeyed by the affinity, equilibrium constant, intrinsic specificity and kinetics for biomolecular recognition. The general statistical laws based on energy landscape theory can serve as a conceptual framework for molecular recognition in biological repertoires. They can be applied to molecular selection, in vitro evolution process, high throughput screening and virtual screening for drug discovery. The statistical laws in combinations with experiments provide quantitative signatures of a specific ligand binding to a specific receptor, these resultant laws as a guideline will contribute to drug design against a specific target. Our developed statistical methodology is general and applicable for all other biomolecular recognitions.

## Introduction

Molecular recognition has been a long standing issue of molecular biology [1, 2]. On the practical side, it is also under intensive investigations in drug discovery and pharmaceutical industry [3]. There are two major issues in biomolecular binding. One is the affinity which is responsible for the driving force and the stability of the binding complex. The other one is the specificity [4, 5] which is crucial for molecular recognition measuring discriminations of “good” binding against “bad” binding. Using microscopic atomistic descriptions to quantitatively study binding is rather difficult [6, 7]. Further more, the accurate estimates of the physical relevant quantities are limited by the delicate balance between hydrophobic interactions, electrostatic interactions, solvation effects and conformational entropy.

The technology advances in combinatorial synthesis of peptide [8] as well as virtual screening of small molecule databases provide a new route [9, 10] to address the above mentioned issue. Instead of studying a specific pair of biomolecular binding, for example, a receptor-ligand (or receptor-small molecule) complex, one can now search through the sequence space of ligands or different small molecules and perform the binding experiments for each specific sequence of ligand binding to a particular receptor. The sequences or segments with good binding properties with the receptor will be the ones to select. Therefore by exploring the sequences of ligands (typically 10^6^ ∼ 10^9^ sequences, a large number which is perfect for the statistical description) or small molecule databases (the number of small molecules can be on the order of 10^60^), one can explore the biological specificity and function by mimicking the natural evolution selection process with the fittest surviving from the ensemble of ligands. The combinatorial library or virtual screening of small molecule database therefore provides a natural laboratory for uncovering the fundamental principles in biomolecular binding and design through the study of the statistical features of the ensembles of the ligands or small molecules with different sequences binding to a specific receptor. In this way, one can characterize the receptor-ligand system and obtain the important statistical information and distributions on the relevant physical properties or observables of the system such as the binding free energy, equilibrium constants, and specificity. The distribution of the physical variables obtained can be universal revealing the common features among different biomolecular binding complexes. It helps the understanding of the evolution and function. The values of the parameters in the distribution characterizing the properties of the underlying energy landscape may be different for different binding complexes. They can be inferred from the experiments.

### Theory and Analytical Models

**Fig 1:**
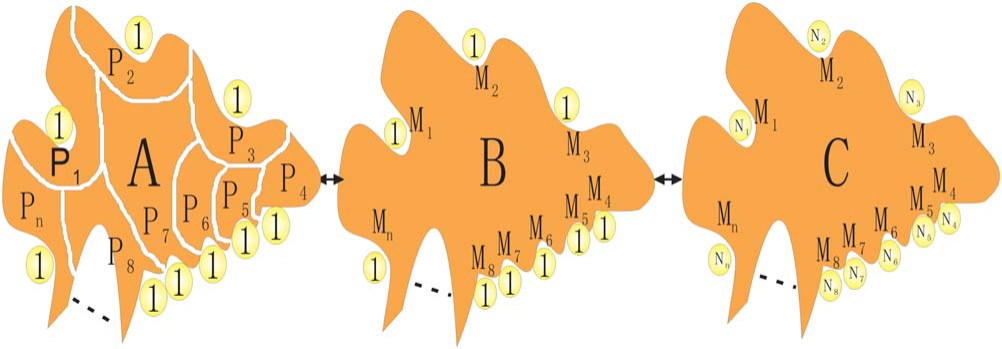
The physical equivalences in probing interactions of molecular recognition. (A) A specific ligand binding to different receptors, *P*_1_∼*P*_*n*_ represent the different receptor proteins with the associated binding sites. (B) Different interactions through different atomic contacts of a specific pair of ligand-receptor complex, *M*_1_∼*M*_*n*_ represent the different interactions with different set of contacts located at the different binding sites of a specific receptor. (C) A specific receptor binding to different ligands, *N*_1_∼*N*_*n*_ represent the different ligands with different sequences.

Experiments on random sequence protein folding [11] and protein design [12], have implied the statistical distributions of physical observable. Experimental and computational studies on biomolecular binding have also shown evidences of distribution of physical relevant quantities such as binding equilibrium constants K [13–18]. Since Log K is proportional to the free energy difference between the native and non-native states termed as the stability or affinity, this infers that the free energy also has a distribution. The experimental features on binding indicate that the appropriate physical variable is the free energy of binding of ligands to the receptor, not the energy. The similar situation also appears for random sequence protein folding and protein design. As a result, the free energy stability or affinity for each specific sequence of ligand can be obtained. Certain values of the affinity appear more often than others. The free energies therefore are distributed. When we discuss about the distribution of the free energies, we mean the sampling of different free binding energies from the different ligands with different sequences binding to the same receptor. The binding free energy of each individual ligand to a specific receptor can be calculated and measured directly from the experiments (through the equilibrium constant measurements). Collecting the free energies from different ligands binding to the same receptor, we can find the distributions of the free energy. The similar procedure applies to the statistics of specificity, equilibrium constants and kinetics for different ligands binding to the same receptor protein. We mainly focus on the statistics of the different ligands binding to the same receptor protein in this study. By exploring the sequence space of ligands, one also equivalently goes through the interactions and conformation space under the ergodicity condition. Here, we describe a physical hypothesis based on a thought experiment (see the Fig 1) [19–21]. Imagine we connect all the different receptors by linkers (for example, connecting N terminus of a protein and C terminus of another by glycines), then the whole universe of receptors now becomes one giant protein. When the receptor protein is large enough, probing the interactions of binding through different parts (binding sites/pockets) of the same receptor protein (panel B) and probing the interactions through the sequences (different receptors in panel A or different ligands in panel C) should be equivalent. The quantitative issue is how large the receptor protein should be in order to see the above mentioned approximate equivalence. Since the protein folds have been estimated to be on the order of a thousand [22], the actual number of the interactions via the atomic contacts of the ligands with the receptors is finite and enumerable. In other words, a large but finite size protein may already contain most of the interactions encountered for ligand binding. Under the assumption of large receptor protein (large enough to effectively represent the sequences of the diverse receptor universe), obviously searching for all the binding sites or pockets of a particular finite size protein is equivalent to searching for the whole universe of the receptors. Therefore, in this case, probing interactions can be reached approximately equivalently by the following three approaches: (1) multiple ligands binding to the same receptor, or (2) multiple receptors binding to the same ligand, or (3) a ligand binding to a receptor exploring the different binding sites (modes). This hypothesis has been tested and validated for specificity of Cox-2/Cox-1 receptor-ligand complexes [21]. In other words, the statistical properties through the exploration of sequence space of different ligands with the same receptor is equivalent of searching through the conformational or structural space for a particular ligand receptor pair. Although the free energy is in general a complicated function of interactions and entropy, combinatorial library of ligands and database of small molecules provide us a great opportunity to study the interactions and underlying principles of binding. In the free energy distribution, the native (strongest) binding state(mode) should appear in the lowest end of the tail where the density of these binding states becomes discrete.

**Fig 2:**
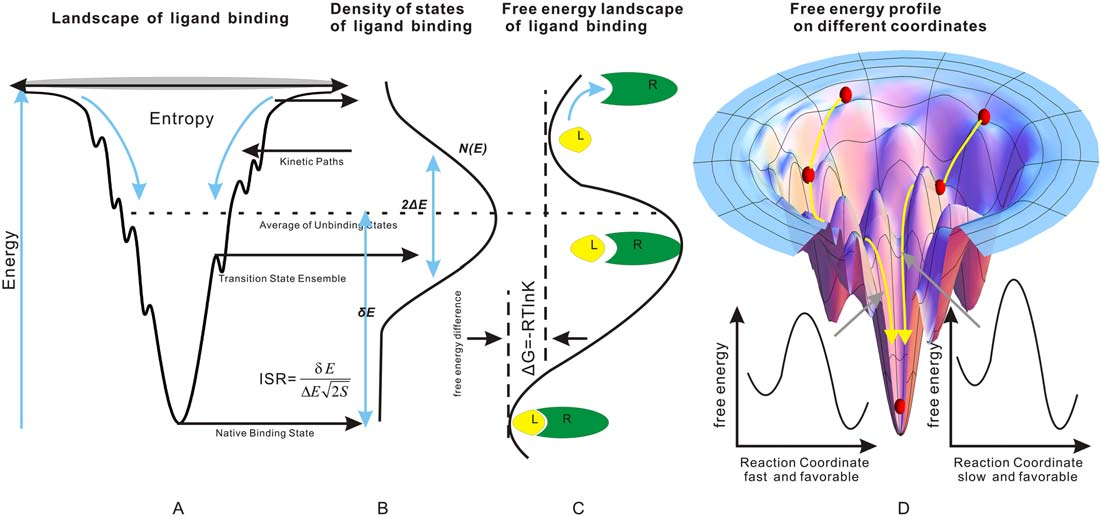
Funneled energy landscape of the biomolecular binding. Panel (A) shows the energy landscape of the receptor-ligand binding with a funneled shape towards the native state. Panel (B) shows the density of states of the ligand binding landscape. The intrinsic specificity ratio $ISR=\frac{\delta E}{2\Delta E\sqrt{S}}$, where energy gap *δ*E and energy roughness ΔE and size of the binding funnel measured by the entropy S are shown respectively. Panel (C) shows the free energy landscape of ligand binding, cartoon showing of receptor/ligand complex corresponds to the different binding states. The affinity measured by the free energy difference between the native binding state and unbound states are shown as Δ*G*. It can also be measured by the equilibrium constant K where Δ*G* = −*RTlnK*. Panel (D) shows the energy landscape and free energy profiles on different reaction coordinates.

The conventional specificity refers to the discrimination of binding affinities between a specific ligand with different receptors. However, it is challenge to go through the whole universe of receptors to determine the binding specificity due to the lack of the full information. From the above equilvalence discussions in terms of probing the interactions of molecular recognition, another way of quantify the specificity is to find the discrimination in binding affinities of a ligand binding with different binding sites of a receptor. This is referred to the intrinsic specificity. Under large protein assumption, the intrinsic specificity and conventional specificity should be equivalent. The supporting evidence was illustrated in the Cox-2/Cox-1 binding with ligands [19–21]. Both affinity and specificity are crucial for determining the molecular recognition. An optimal quantitative criterion for the intrinsic specificity in discriminating the native from the non-native states for the best binding sequences is found to be the maximization of the intrinsic specificity ratio (ISR) of the energy gap between the energies of the native state and the average of the other states versus roughness or fluctuation modularized by the entropy or size of underlying binding energy landscape [19, 20], see panel B of Fig 2 for the density of states of binding. This implies that the underlying binding landscape is funneled towards the native state. This is not surprising since the driving force of the binding is the same as folding, the hydrophobic interactions. The folding can be seen as self binding and binding can be viewed as folding with multiple domains without the linkages between the domains. Therefore, one expects that the resulting binding energy landscape should have a funneled shape towards the native state to guarantee the stability and intrinsic specificity as well as the kinetic speed of recognition against the bumps or wiggles along the binding paths. (see panel A,C of Fig 2). This is in parallel to the protein folding studies where similar optimal criterion for folding has been used to design fast folding protein sequences [23–25]. Different ligands or small molecules will have different specificity for binding with a specific receptor. Therefore, the intrinsic specificity(ISR) should also have a statistical distribution. This reflects different degrees of binding specificity. There is a small group of high specificity ligands among all the available ones. This is rare and lies in the high end tail of the distribution of specificity.

There is a topologically unique native binding state at the bottom of the funnel (See Fig 2). We can use RMSD as order parameter to describe the position of an ensemble of states in the landscape. Through the RMSDs, the binding landscape is stratified and simplified. We can define an average energy over many states with different energies and RMSDs within each stratum. Then the local kinetics between the binding states with the different average energies can be explored, the global kinetics between the native state and non-binding ones can also be described. In other words, the kinetics of ligand receptor recognition on the binding energy landscape can also be studied. We have previously performed analytical studies of kinetics with both global and local connectivity [26, 27] as well as microscopic flexible docking studies of ligand receptor binding [21]. We also found that intrinsic specificity quantified by ISR not only guarantees the thermodynamic stability and specificity but also guarantees the kinetic accessibility and the speed of the recognition. While the average kinetics has been investigated, there are only limited studies on the statistics of the kinetics, mostly at the analyatical level [27–32].

The theoretical and modeling studies on the statistical distributions of the physical variables such as the affinity, equilibrium constants, specificity, and kinetics to uncover the underlying processes and the associated energy landscape of binding and conformational dynamics are currently far less explored than their counter part, the mean. Thus it is the purpose of this paper to fill the gap. Furthermore, the advances of combinatorial chemistry with the capability of generating large number of small molecules at once and high throughput screening provide us a great opportunity of obtaining the statistical information on thermodynamics and kinetics for molecular recognition through the exploration of the ensemble of sequence space of small molecules (combinatorial ligand binding). The statistical information on thermodynamics can be probed from the experiments by measuring the thermodynamic stability through equilibrium constant measurements for different ligands binding with a receptor. On the other hand, the statistical information on the kinetics (on/off) can be probed from the experiments by measuring the kinetic rates through dissociation constant measurements for different ligand binding with a receptor. These statistical information will be crucial to uncover the global and universal features of the thermodynamics and kinetics of the associated molecular recognition. Characterizing the shapes of the statistical distributions of these physical variables relevant to the thermodynamics and kinetics not only allows a better statistical description of the experimental and theoretical results but also permits the testing of hypothesis about the underlying molecular recognition processes. It is also important for the practical application of drug discovery.

## Results and Discussion

We will first present the analytical results and then the simulation results. For analytical studies, since we are mostly interested in the general laws governing the biomolecular binding, the task here is to obtain the universal features. We can use the coarse grained level description instead of the microscopic detailed one to characterize the system.

The Hamiltonian or energy function for the interactions between ligands and receptors can be described by the collections of contact interactions between the atom pairs *E* = ∑_*ij*_ *J*_*ij*_ *σ*_*ij*_, where *σ*_*ij*_ is the contact variable between atoms i and j with certain distance cutoff and the *J*_*ij*_ is the coupling strength for specific contact pair between i and j. Since there are many different types of atoms and also many different cutoff distances for the interactions, different *J*_*ij*_ can have different values. This forms a distribution for the coupling strength J. Since the number of different J couplings are large, the statistical distribution should have a Gaussian form from the large number theorem. Since the energy is linearly related to the coupling. This leads to a random energy model with the interaction energy follows a Gaussian distribution [19, 33, 34]. This reflects the complexity of the underlying interactions in contrast to the conventional deterministic models for simple systems where the coupling strengths are fixed and not distributed [35]. Furthermore, we can study the energy landscape of ligand binding using the random energy model with the certain bias towards the native state [19]. This assumes that the energies of non-native states and their interactions are random variables with given probability distributions as discussed above. The binding process can be illustrated as random walks on a rough binding energy landscape with bias towards the native state [19]. Based on the more detailed description in S1 Text [26–30, 32, 36, 37] and the simulation method of this study, we can obtain the free energy, equilibrium constant, intrinsic specificity and the kinetics distribution from the random energy model analytically and those from the direct simulations.

### Analytical Models of Distribution of Affinity, Equilibrium Constants, Specificity and Kinetics

#### Universal distribution of free energy of biomolecular binding

Because the underlying interaction energy is Gaussian distributed, the functional form of the distribution of the resulting free energy can be obtained by carefully studying the moments of the partition function of the resulting random energy model [36, 37]. The distribution function f has the form of:

$$\begin{array}{c} f\left(F\right)∼exp\left[-\frac{{\left(F-\bar{F}\right)}^{2}}{2\Delta E^{2}}\right] \end{array}$$ (1)

where f(F) is a Gaussian distribution of the free energy F around its mean $\bar{F}$ (See details in S1 Text) with the variance of the distribution Δ*F* ^2^ = Δ*E* ^2^ (The width of the energy distribution Δ*E* here has the meaning of the roughness of the underlying energy landscape above the trapping transition temperature *T*_*c*_. $T_{c}=\sqrt{\frac{\Delta E^{2}}{2S}}$ is the trapping transition temperature representing the onset of the local trapping of the underlying energy landscape, S represents the configurational entropy measuring the size of the configurational space which scales with the size of the system (number of atoms), herein, using the ligand-receptor binding complex as a system. S = *K*_*B*_logΩ where Ω represents the number of states in the configurational space.) Furthermore, f(F) can be shown to have the exponential distribution in the tails near or below *T*_*c*_ [32, 36, 37]:

$$\begin{array}{c} f\left(F\right)∼exp\left[\frac{\rho (F-F_{c})}{T}\right]\theta \left(F_{c}-F\right) \end{array}$$ (2)

where $\rho =\frac{T}{T_{c}}$ and *F*_*c*_ is the cut-off free energy(*θ* function is defined as *θ*(*x*) = 1 when *x* > 0;*θ*(*x*) = 0 when *x* < = 0). Notice that the mean free energy is close to ∣*F*_*c*_ − *T*_*c*_∣ (here, *K*_*B*_ = 1) and the width of the distribution is on the order of *T*_*c*_. Notice that the distribution of free energy develops an exponential tail which decays slower than the Gaussian distribution. Therefore, the rare (low probability) events with low free energies in the spectrum of the specific sequences are more prominent in the exponential distribution.

**Fig 3:**
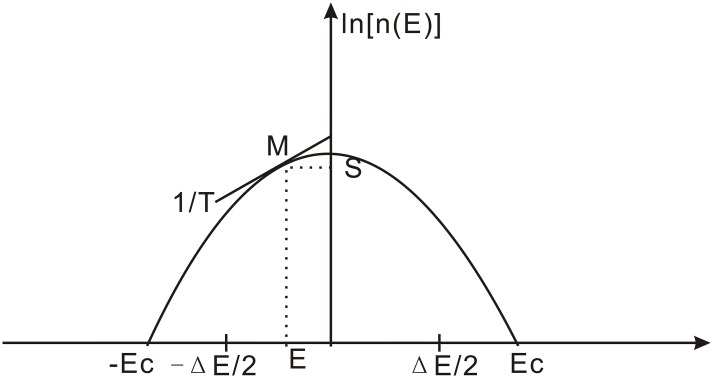
The entropy S(E) as a function of energy for the random energy model. The curve is entropy S(E) = ln[n(E)], where n(E) is the density of states with energy E from the spectrum, *E*_*c*_ is the corresponding energy of the trapping transition temperature *T*_*c*_. As we see, the point M moves on the curve with the changes in the slope (1/T).

It should be noted that in the aforementioned analytical random energy model [33, 38, 39], from the thermodynamic Maxwell relation of *δ*S(E)/*δ*E = 1/*T*(Fig 3) where *S*(*E*) is the entropy of the system. Since entropy is related to the distribution of the states in energy or density of states (*S* = *ln*[*n*(*E*)]), we can see the relationship between the energy (left side of the equation) and temperature (right side of the equation). Physically it is clear that the slope of distribution is inversely proportion to temperature (Fig 3). In other words when the temperature is high (the slope of the distribution 1/T is low), then the energy is at the center of the distribution and when the temperature is low (slope of the distribution 1/T is high), then the energy is at the tail of the distribution (Fig 3). This can be seen clearly in Fig 3. Since free energy is related to energy, high energy leads to high free energy. Therefore, when temperature is high, then the free energy is at the center of the distribution and when the temperature is low, then the free energy is at the tail of the distribution (Fig 3). This is also true for the affinity, equilibrium constant, ISR and kinetic rate distributions where all of these variables are directly related to the free energy. Therefore, these give the correspondences of the analytical results for distribution of the variables in different temperature ranges with the simulation results for distribution of the variables in different variable ranges. On the other hand, since partition function Z has an asymptotic form and a power law distribution at the tail, the associated free energy is therefore exponentially distributed at the tail (x→∞)(See details in S1 Text). In other words, for the distribution of free energy, we can obtain two distinct regions (such as low and high free energy) of the corresponding physical variable.

As one can see, it is essential to not only know the information about the mean, but also at least the width of the distribution, in order to characterize the whole free energy spectrum. Failure to do so results incomplete information of the system. It is interesting to notice that experimental evidence for the distribution of free energy seems clear. However, the average is usually obtained and used to study the thermodynamics and the kinetics in the current microscopic atomistic simulations on the binding, where the free energy perturbation and other approximation methods are often used. The higher order statistical information such as variance and the whole distribution are not noticed with much attention and hardly explored. As we point out, with the computer hardware speed rapidly improving, the more comprehensive information can be obtained in silico. Careful analysis based on microscopic simulation studies, the determination of the parameter values appearing in the current analytical approach and comparisons with the experiments are crucial for the more complete description of the binding process.

#### The universal distribution of the equilibrium constant of biomolecular binding

The equilibrium constant K for the biomolecular binding complex measured often in the experiments is defined as the difference between the free energy of the native *F*_*n*_ and the completely non-native states *F*_*un*_:$logK=\frac{F_{n}-F_{un}}{RT}$. Note that as the differences of the free energies of the native state relative to the non-native states *F*_*n*_ − *F*_*un*_ decreases to more negative values, the stability increases. The Log K decreases too. The biomolecular binding complex is more stable. The affinity also increases. Therefore from the definition, the Log K measures the ability of the two biomolecules associate with each other or the affinity. The equilibrium constant can be measured directly from the experiments. In the combinatorial experiments, one can further measure the statistical information of the equilibrium constant by exploring different ligand binding with a receptor one at a time. Based on the probability theory and statistics, if the random variable X is log-normally distributed, then Y = log(X) has a normal distribution. Likewise, if Y has a normal distribution, then X = exp(Y) has a log-normal distribution. In other words, by knowing the distribution of free energy, one can derive the probability distribution of the equilibrium constant K ($logK=\frac{F_{n}-F_{un}}{RT}$) as well since the logarithm of the equilibrium constant K is directly related to the free energy difference between the native and non-native states (the non-native states are Gaussian distributed while the native state is very narrowly distributed). Since the aforementioned distribution of free energy is the Gaussian in this study, one can easily obtain the distribution function of the equilibrium constant K as log-normal. So, the distribution function f of the equilibrium constants K or the affinity can be shown to be:

$$\begin{array}{c} f\left(K\right)∼\frac{1}{K}exp\left[-\frac{T^{2}}{2\Delta E^{2}}{\left(LogK-Log\bar{K}\right)}^{2}\right] \end{array}$$ (3)

which is a log-normal distribution near the mean above *T*_*c*_ while

$$\begin{array}{c} f\left(K\right)∼K^{-1-\frac{T}{T_{c}}} \end{array}$$ (4)

which shows a power law decay near the low K value tail of the distribution (near or below *T*_*c*_).

This power law behavior is familiar in the physics and chemistry community as a signature of the long range order and self similarity often appeared in the critical phenomena of phase transition, fractals, turbulence and earth-quakes etc. The slow power law decay (as compared with the Gaussian like distribution) indicates a long tail in the equilibrium constant distribution. In this situation, similarly, the average is no longer a good representative of the distribution. The whole distribution function is needed to characterize the system. This implies that the rare events at the tail may play a dominant role. The phenomenon is often termed as intermittency. The power law behavior of the equilibrium constant in the tail therefore indicates that most of the binding molecules show small binding affinities, only occasionally a specific pattern will lead to high affinity. Therefore, characterizing the global shape of the statistical distribution of equilibrium constant is necessary for the molecular binding. Finding high affinity or the associativity is rare but important to study the evolution and function for binding. By obtaining the experimental measurements of the distributions one can estimate the relevant thermodynamic quantities that fit the experiments from more microscopic approaches based on molecular interactions and structures. Comparing model distributions here with the experiments may also guide the approaches to uncover the underlying correlations between native binding free energy and affinity (equilibrium constant).

#### The intrinsic specificity and its universal distribution for biomolecular binding

From the distribution of the free energy studied in this paper $f(F)∼exp\left[-\frac{{\left(F-\bar{F}\right)}^{2}}{2\Delta E^{2}}\right]$, we see that the width or the fluctuations of the free energy spectrum Δ*F* is equal to Δ*E*, and the average of the free energy is $\bar{F}$. As discussed earlier, affinity giving the ability of association of two molecules in general can not guarantee the intrinsic specificity that gives the discrimination of the native state from non-native ones. To select a good sequence for binding and realize the specificity, one should have the free energy gap between the ground state or the cluster (group) of states with the lowest free energy of binding relative to the average free energy to be large compared with the width of the distribution of the binding free energy. Only in this way one can discriminate the native state from others and guarantee the intrinsic specificity. That is:

$$\begin{array}{c} Maximize\frac{|F_{n}-\bar{F}|}{\Delta F} \end{array}$$ (5)

where *F*_*n*_ is the free energy of the native state located in the low energy tail of the distribution. This gives the optimal criterion of binding specificity. If we consider further the detail of the exponential distribution near the tails: $f(F)∼exp[\frac{T}{T_{c}}\frac{\left(F-F_{c}\right)}{T}]$ where *F*_*c*_ as previously mentioned is the cutoff free energy, the mean is equal to *F*_*c*_ − *T*_*c*_ and the width is equal to *T*_*c*_, the optimal criterion for specificity now becomes the maximization of $\frac{\left|F_{n}-(F_{c}-T_{c})\right|}{T_{c}}$.

From the expression of the free energy distribution, one can easily obtain the distribution of the native free energy to be: *f*(*F*_*n*_|*N*) = *f*(*F*_*n*_)*f*(*F*_*n*_ < *N*_*t*_ − 1) where *F*_*n*_ is the native free energy, $f(F_{n})∼exp\left[-\frac{{\left(F_{n}-\bar{F}\right)}^{2}}{2\Delta F^{2}}\right]$ is the probability of finding the free energy *F*_*n*_ and $f\left(F_{n}<N_{t}-1\right)={\left[\overset{\infty }{\underset{F_{n}}{\int }}f\left(F\right)dF\right]}^{N_{t}-1}$ is the conditional probability of free energy of native state less than the rest of the spectrum assuming there are total *N*_*t*_ number of states [40, 41]. Based on this, one can easily convert the above distribution of the native state free energy distribution to the one for the specificity *ISR*, ($ISR=\frac{\left|F_{n}-\bar{F}\right|}{\Delta F}$). That is:

$$\begin{array}{c} f\left(ISR\right)∼exp\left[-\frac{ISR^{2}}{2}\right]{\left[\frac{1}{2}\left(1+Erf\left(\frac{ISR}{\sqrt{2}}\right)\right)\right]}^{N_{t}-1} \end{array}$$ (6)

near the mean and above the trapping temperature *T*_*c*_. The distribution becomes narrower and the center of the distribution shifts towards larger values of *ISR* when the number of states is larger. In addition, the distribution becomes more skewed under this situation. This implies that as the number of states become more and more, averagely speaking, there are more and more chances of finding the more stable complexes with larger values of *ISR*. The corresponding distribution *ISR*($ISR=\frac{\left|F_{n}-(F_{c}-T_{c})\right|}{T_{c}}$) near the tail around or below *T*_*c*_ can be shown to be:

$$\begin{array}{c} f\left(ISR\right)∼exp\left[-ISR-1\right]{\left(1-exp\left[-ISR-1\right]\right)}^{N_{t}-1} \end{array}$$ (7)

Therefore there is exponentially small probability of finding large values of *ISR* although the actual absolute number of ligand sequences with large *ISR* can be large. If one makes a histogram for the values of the specificity *ISR* for the native states and random sequences, one would expect to see the center of the two distributions are well separated by a gap which guarantees the intrinsic specificity. Therefore one can use the optimal criterion to select the stable and specific complexes with good and discriminative binding properties from random sequences.

#### Kinetics and its universal distribution for biomolecular binding

Under the quasi-equilibrium condition, the time scale *τ* of binding of a particular sequence of ligand with a receptor can be approximated as: $log(\frac{\tau }{\tau_{0}})=\frac{F^{♯}-F_{un}}{T}$[28, 29]where *F* ^♯^ is the free energy of the transition state ensemble, *F*_*un*_ is the free energy of the completely non-native states, *τ*_0_ is prefactor for the time scale for the binding process between binding states. Therefore, the time scale of binding from one binding state to a neighboring state is determined by the free energy difference of these two states. In this study, the first passage time(FPT) to reach the native binding state(the time required for the random walker to visit order parameter RMSD∽0 for the first time) is used as a typical or representative time scale for binding. The mean first passage time(*τ*) representing the binding time has a U(or V) shape dependence on temperature [27]. Similar to the behavior of K, assuming a common *F* ^♯^, the distribution of *τ* can be shown to have the form as:

$$\begin{array}{c} f\left(\tau \right)∼\frac{1}{\tau }exp\left[-\frac{T^{2}}{2\Delta E^{2}}{\left(log\frac{\tau }{\tau_{0}}-log\frac{\bar{\tau }}{\tau_{0}}\right)}^{2}\right] \end{array}$$ (8)

giving log-normal distributions near the mean above *T*_*c*_ while

$$\begin{array}{c} f\left(\tau \right)∼\tau^{-1-\frac{T}{T_{c}}} \end{array}$$ (9)

gives much slower power law decay distributions near the tails of the distribution near or below *T*_*c*_) [31, 32].

Previously we analytically explored the origin of power law distribution of kinetics observed in single molecule conformational dynamics experiments [28, 29, 32]. We established a diffusion and a kinetic master equation approach to study statistically the microscopic state dynamics. The exponential density of states emerges when the system becomes glassy and landscape becomes rough with significant trapping under low temperatures as shown earlier from the analytical studies [32, 36, 36, 37]. We show that the underlying landscape with exponentially distributed density of states leads to power law distribution of kinetics(*f*(*x*) ∼ *τ* ^−1 −*T*/*Tc*^). We predicted that the power law decay coefficient is monotonically dependent on temperature which can be tested from ongoing experiments. This may bridge statistics from single molecule kinetic experiments and topography of conformational energy landscape [42–48].

Therefore, there is a physical explanation of the difference in distributions of binding kinetics: Above the characteristic transition temperature *T*_*c*_, there are multiple parallel kinetic paths, each experiencing certain barriers (barrier has a normal distribution), resulting in log-normal kinetics (seen also in protein conformational dynamics simulations [31]). When the temperature drops below *T*_*c*_, the distribution of FPT deviates from log-normal significantly. The traps become more important. Discrete pathways emerge. The distribution at long times approaches a power law. This indicates that the fluctuations in FPT are significant and deviate from the mean [27]. The fluctuations start to diverge. This means that the actual conformation dynamic process may happen on multiple time scales, and the nonself-averaging behavior emerges. The distribution of FPT then has fatty tails. This indicates that the intermittent phenomenon, where rare events can give a significant contribution to the binding. Due to the ruggedness of the binding energy landscape at low temperatures, specific different discrete paths give distinct contributions to the kinetics. The shape of distribution of FPT is then required to characterize the whole system dynamics rather than only the mean.

It is worthwhile to emphasize that our analytical studies in conformational dynamics, protein folding and ligand binding all indicate that the kinetics should follow log-normal distribution at higher temperatures and power law distribution at the low temperatures [27–29, 31, 32]. Our analytical studies formed a mathematical foundation and motivation for us to explore the distribution in kinetics in real ligand binding.

Notice that due to the equivalence of the ensemble exploring different ligands and ensemble exploring different interactions through different atomic contacts of specific ligands to receptor, the statistical distribution of the physical relevant quantity such as binding affinity from different ligands binding to the same receptor is equivalent to the distribution of the one obtained from different interactions of the specific ligand-receptor binding interactions through spatial atomic contacts(See Fig 1). Therefore in analytical model, for example, we can obtain the distribution of binding affinity for the ensemble of different atomic contact interactions (with different non-native unbinding states). Due to the equivalence, this implies that the affinity for receptor binding to different ligands or ligand binding to different receptors is expected to follow the same statistical distribution.

### Microscopic Atomic Binding Model and Simulation Results

**Fig 4:**
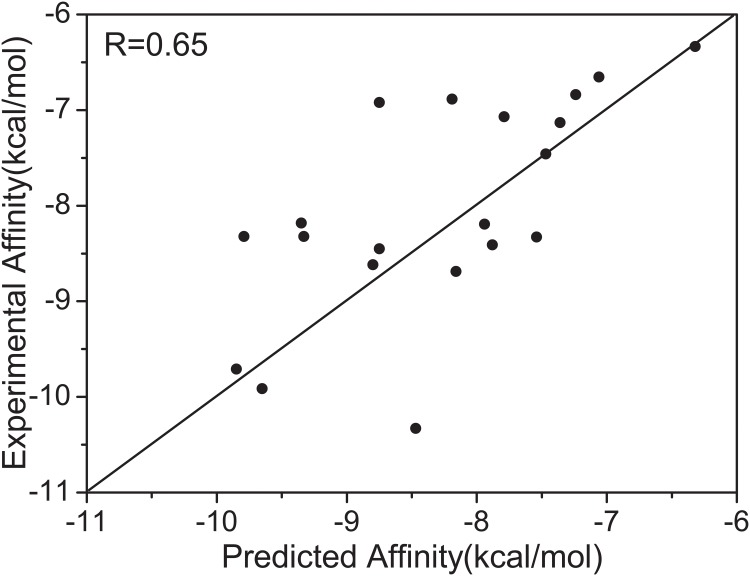
Validation of Autodock scoring to predict the binding affinities for 20 drugs against the Cox-2.

We have performed the investigation of the significance and implications of flexible docking of ligands with the receptor target COX-2. Initially a diverse set of 720 small molecules were selected from the NCI-Diversity database [49] having molecular weights similar to that of the reference compound SC-558, for which the crystal structure of the COX-2 complex is available (PDB code 1CX2) [50, 51]. All conformers of each of the 720 selected molecules were docked with COX-2 using AutoDock [52] to generate a binding energy spectrum for each. Furthermore, the correlation coefficient of 0.65 between experimental and predicted affinities (Fig 4 and S1 Table) demonstrate reasonable reliability of Autodock scoring for the Cox-2 target.

**Fig 5:**
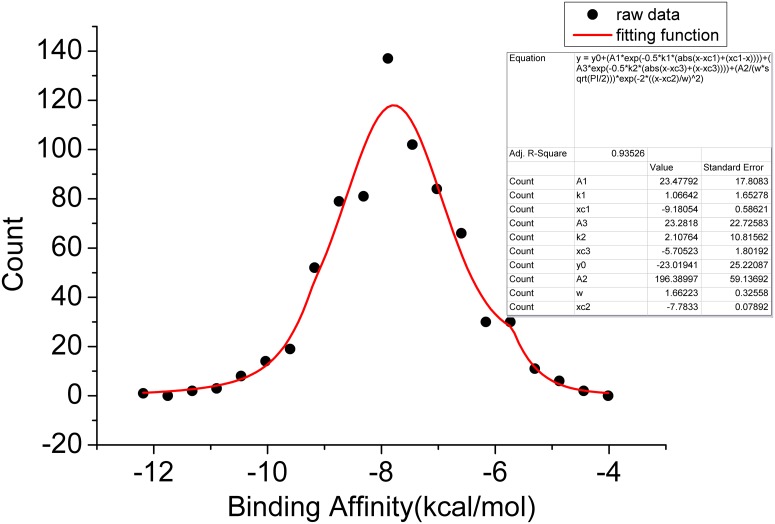
The distribution of the free energy with 720 compounds binding to the Cox2. the vertical axis represents the number of states or probability for the free energy, gaussian curve: in the center; exponential curve: near the tail. The parameter values for these distributions are presented. The analytical function consisting of a Gaussian and two exponential functions is highlighted with red line.

From this data, the statistical distribution of the affinity (defined as the free energy difference between the native state and the average of the free energy) of 720 small molecules with COX-2 was described. Fig 5 shows the distribution of affinity. As easily seen, near the center or the mean, the distribution can be fitted well with a Gaussian. Near the tail, the distribution of the affinity can be fitted well with exponential. This confirms the analytical results discussed above. Most of the ligands bind to a receptor with relatively small affinities. A small number of the ligands has high affinity to a receptor, these ligands are crucial for exploring the biological function of the receptor.

**Fig 6:**
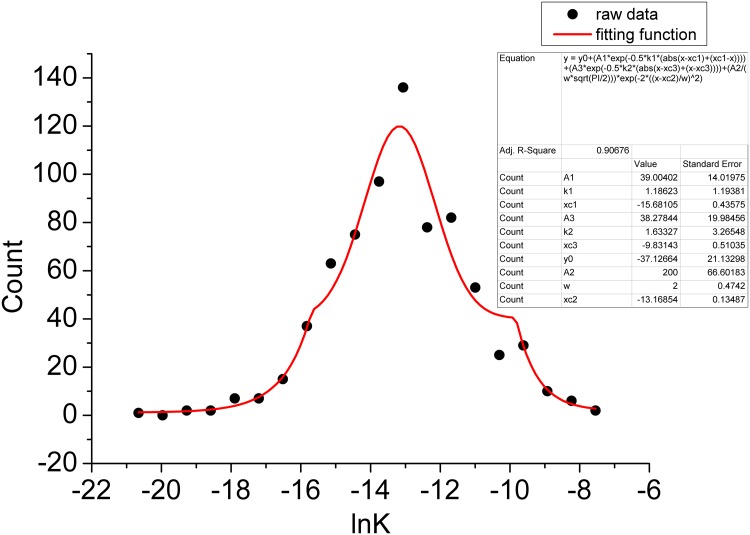
The distribution of the logarithm of equilibrium constant with 720 compounds binding to the Cox2. the vertical axis represents the number of states or probability for the equilibrium constant, gaussian curve: in the center; exponential curve: near the tail. The parameter values for these distributions are presented. The analytical function consisting of a Gaussian and two exponential functions is highlighted with red line.

In Fig 6, we show the logarithm of the equilibrium constant distribution for the 720 small molecule binding with Cox2. We see that the logarithm of equilibrium constant can be fitted well with a normal distribution near the mean while near the tail can be fitted well with a exponential distribution. Thus, we can extrapolate the statistical properties of the equilibrium constant distribution. That is, the equilibrium constant can be fitted well with a log-normal distribution near the mean while near the tail can be fitted well with a power law distribution. In contrast to the gaussian distribution, the log-normal distribution has a longer tail representing the higher frequencies of equilibrium constant when plotted in the original scale without taking the logarithm. The power law distribution of the equilibrium constant suggests most of the equilibrium constants of the ligands with random sequence binding to the receptor are small and binding complex are less stable. The large equilibrium constants although rare can contribute greatly in determining the biological stable function. They are the critical ones in molecular recognition.

**Fig 7:**
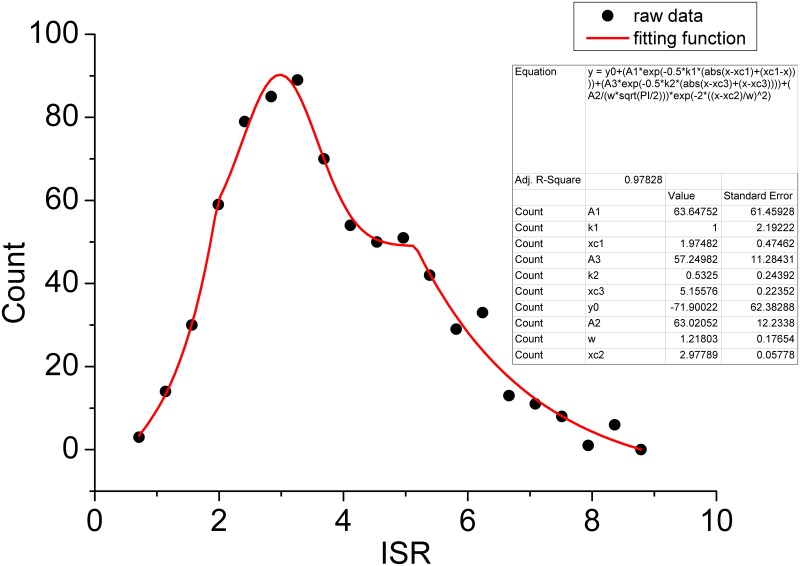
The distribution of the specificity(ISR) with 720 compounds binding to the Cox2. the vertical axis represents the number of states or probability for the specificity, gaussian curve: in the center; exponential curve: near the tail. The parameter values for these distributions are presented. The analytical function consisting of a Gaussian and two exponential functions is highlighted with red line.

In Fig 7, we show the statistical distribution of the intrinsic specificity characterized by ISR. We also see that the distribution of the intrinsic specificity can be fitted well with the normal distribution near the mean and also well fitted with the exponential distribution at the tail. This means most of the ligands with random sequence non-specifically bind to a receptor with low specificity. A few ligands specifically bind with a receptor with high specificity. They are at the high end tail of the intrinsic specificity distribution.

**Fig 8:**
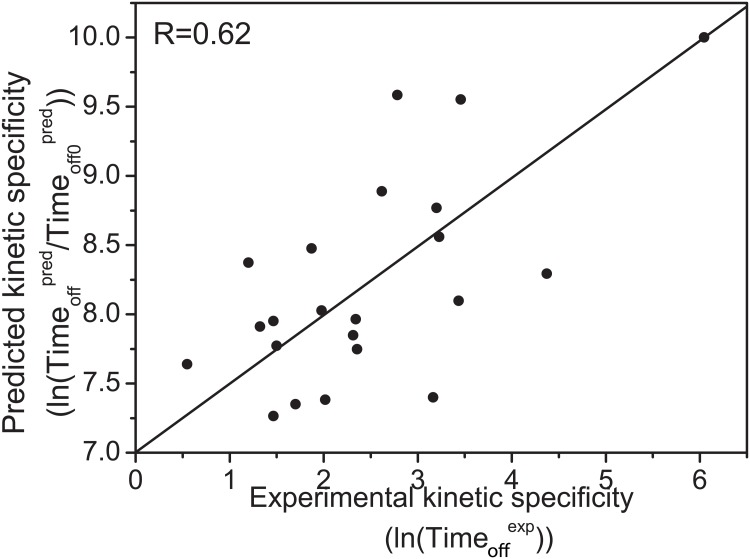
The relationship between the predicted and experimental kinetic specificities for 22 drugs against the Cox-2. $Time_{off}^{pred}$ represents the relative predicted residence time and *Time*_*off*_0__^pred^ is the constant weighting factor, $Time_{off}^{exp}$ is the experimental residence time.

Herein, we have computationally determined the kinetic specificity of binding using kinetic off time of binding *Time*_*off*_ to represent the time scale *τ*. To demonstrate the reliability of the *Time*_*off*_ we have defined, the analysis regarding the correlation between the predicted and experimental kinetics have been performed for the 22 drugs against the Cox-2 target, a reasonable correlation with the coefficient 0.62(Fig 8 and S1 Table) was obtained.

**Fig 9:**
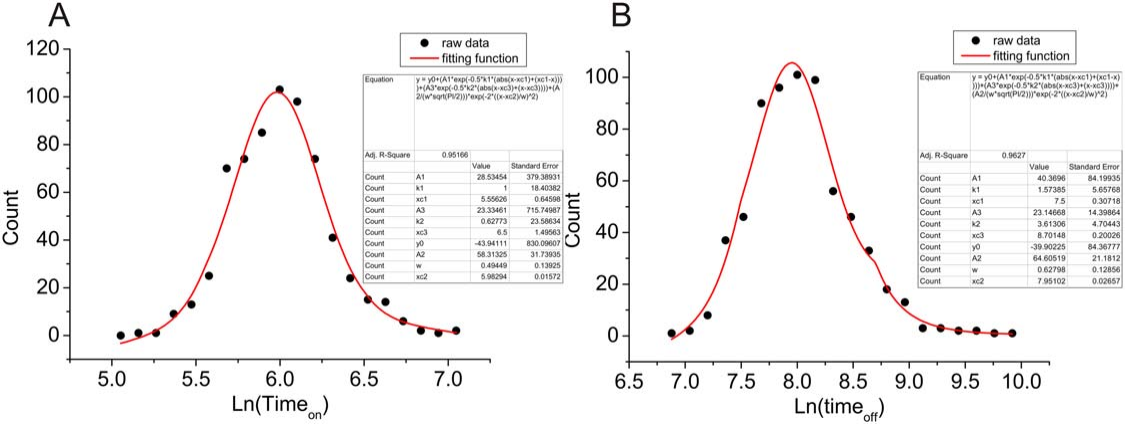
The distribution of the predicted kinetic specificity. **(A)** The distribution of the logarithm of the predicted *Time*_*on*_ with 720 compounds binding to the Cox2; **(B)** The distribution of the logarithm of the predicted *Time*_*off*_ with 720 compounds binding to the Cox2. gaussian curve: in the center; exponential curve: near the tail. The parameter values for these distributions are presented. The analytical function consisting of a Gaussian and two exponential functions is highlighted with red line.

As described in Fig 9, we found that the distribution of kinetics can be fitted quite well with log-normal distribution near the mean and power law distribution at the tail. This is consistent with our analytical models above.

The affinity and equilibrium constants can be measured by experiments directly. From combinatorial point of view, each ligand binds to a receptor with certain affinity and equilibrium constant. By collecting the measurements of different ligands (combinatorial chemistry of peptides or screening with a library of small molecules) each binding with a receptor, average of these quantities can be obtained. Furthermore the statistical information such as high order moments and distribution (histograms) or probability can be also obtained. Similarly, the kinetics can be also explored easily for the statistical information. Intrinsic specificity, on the other hand, is quite difficult to directly measure. However one can indirectly infer the statistical information for the intrinsic specificity from the statistical measurements of both the affinity and kinetics. The ligands with the same affinity might still have different specificity from kinetic point of view.

It is important to realize that although the functional form of the distribution is universal from one ligand to another, the fitting coefficients or parameters in the distribution are different for each ligand. This gives a quantitative signature or characterization of a specific ligand binding to a specific receptor. Importantly, we have found analytical functional expressions for the distributions of free energy, specificity, equilibrium constants and kinetics covering the whole range of these variables including near the mean and at the tail as well as the regime in between (See details in S2 Text) which can give Gaussian statistics at the center and exponential statistics at the tail, respectively.

**Fig 10:**
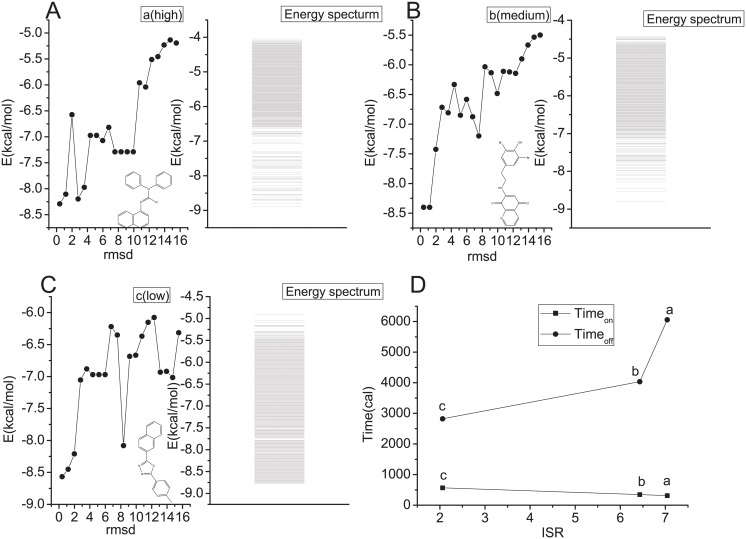
Three representative ligands analyzed in the docking simulations. The left panels of **(A)**, **(B)** and **(C)** show the one dimensional projection of binding free energy landscape to RMSD with high, medium and low ISR with similar affinity as well as the corresponding structures of the different ligands, where the docked pose with the most stable affinity or the strongest binding state is chosen as the reference structure to calculate the RMSDs; the Autodock score function is used to evaluate the interaction energies between the ligand and the receptor. (high:fork structure, medium:near-linear structure, low:linear structure);the right panels of them show the corresponding binding energy spectrum for each, the sparse part of the spectrum implies there are fewer states, and dense part of the spectrum implies there are more states. **(D)** show the kinetic time for binding for the above three different ISRs. The upper black line represents the predicted kinetic time_*off*_ and the under black line represents the predicted kinetic time_*on*_. The vertical axis represents the calculated kinetic time.

In Fig 10A–10C, we also show the one dimensional projection of binding free energy landscape to RMSD with different ligands with different intrinsic specificity characterized by ISR. Different free energy landscapes give different free energy barrier heights between the non-native and native states. This leads to different kinetics for binding(Figs 2 and 10D). As we can see the large intrinsic specificity characterized by high ISR gives smaller free energy barrier and lower ISR gives higher barrier. There is also a structural correspondence shown in the figure for different ligands. The ligands with the fork structure have high structural specificity and faster kinetics, and ligands without the fork structure have low specificity and slower kinetics. We plot the free energy barrier height and the kinetic time for binding of these three cases as shown in Fig 10D. By exploring the different off rates *Time*_*off*_ and on rates *Time*_*on*_ of binding for three ligands, we found that the ligand with higher ISR has faster on rate and slower off rate. In other words, the ligand has longer residence time for binding. In contrast, the ligand with lower ISR has slower on rate and faster off rate with shorter residence time. In a word, we found indeed that the large ISR leads to lower barrier height and faster kinetics. This implies that the kinetics is determined by the intrinsic specificity which is determined by the competition of the slope of the landscape towards the native state and the roughness of the landscape, not just by the affinity alone as conventionally people would have thought. There are recently growing experimental evidences of the similar affinity but completely different kinetic accessibility [53, 54].

In the experiments, we can have a quantitative indicator through kinetics for specificities from each ligand binding to the receptor. It is also interesting to note that large ISR tends to imply the underlying energy landscape is a smooth funnel towards the native state and binding kinetics is fast. This usually results exponential kinetics. The smaller values of ISR tend to imply bumpy energy landscape with the kinetic rates following a slow decay distribution, producing non-exponential kinetics.

The non-covalent interactions in biomolecular recognition have been extensively analysed. HTS(High Throughput Screening) and VS(Virtual Screening) technologies which are pertinent to receptor and ligand repertoires, are increasingly becoming significant for exploring the universal statistical behavior of the entire ensemble of binding. This may provide us an insight to the physical universal laws that underlie non-covalent interactions in biomolecular recognition. An intriguing result of the present distribution analysis is the possibility of extrapolation of the fitted curves from the center to tail region(for example, the low affinity and the high-affinity domain). Thus, based on the fitted parameter from relatively small ligand database (about 700 members in the current study), which statistically attain binding affinities in the range of -4kcal/mol to -12kcal/mol; it is possible to derive information regarding the much higher binding affinities for larger libraries. The probability and physical relevant quantity range is found to fit adequately the distribution functions from the analytical model, and the two kinds of curves are fitted well to the data in the center and near the tail.

Based on its ability to describe the physical relevant quantities and patterns over the different range, from in the center to near the tail, the general distribution laws based on the analytical model from the energy landscape theory can serve as a conceptual framework to explore molecular recognition in biological repertoires. It could be naturally applied to study human olfactory threshold variability [13, 55], autoimmune phenomena such as self vs. non-self recognition [56–58], and specificity of drugs for the Cytochromes P450 repertoire. Remarkably, the comprehensive knowledge of the statistics of affinity distributions in many studies have led to a better understanding of molecular selection [59], and in vitro evolution process such as combinatorial library panning, SELEX technology exploring the RNA/DNA-ligand interactions [60–64], and high throughput screening for drug discovery to study the receptors or enzymes binding with the lead compounds [65, 66].

### Summary and Conclusion

We have discussed the main statistical features of binding free energy landscapes. In particular we obtained the analytical functional form of the probability function of the binding free energy to be Gaussian distributed near the mean and exponential-like distributed in the tail. Furthermore we obtain the log-normal distribution near the mean and power law decay distribution near the tail for the equilibrium constants of binding. The optimal criterion of native ground state dominance and the intrinsic specificity should be the maximization of the ratio of free energy gap between native state and the average non-native states versus the width of the distribution of the free energy landscape. The quantitative form of intrinsic specificity criterion can be used to develop new algorithms to design ligands with high intrinsic specificity for a particular receptor. Alternatively, one can design receptors that are selective for a particular ligand. The statistical distribution of the intrinsic specificity ratio is also Gaussian distributed near the mean and exponentially distributed near the tail. We give an analytical form of the distribution of the kinetics. We have confirmed the analytical form of the distribution functions with 720 diversified small molecules binding with a specific receptor: Cox-2 protein enzyme.

We have found there are universal statistical distributions of affinity, equilibrium constant, intrinsic specificity and kinetics of ligand binding. Furthermore, the coefficients and parameters characterizing the ligand binding are different for each ligand. It is worthwhile to point out that there is a weak positive relationship between affinity and intrinsic specificity. We also found a positive correlation between affinity and kinetics of ligand binding(See S1 and S2 Figs). Molecular recognition in protein-ligand, protein-protein and protein-DNA interactions often involves interplay between affinity and specificity or thermodynamics and kinetics. As known, high affinity does not always guarantee the high specificity. We can see that the correlation between affinity and intrinsic specificity is weak because there is a large spread in the correlation plot between the two. That is to say there is a large dispersion in intrinsic specificity at given affinity (S1 Fig). In general, we need both affinity and specificity to quantify the molecular recognition process. For the lead compound search in drug discovery, high specificity is often achieved by increasing the specific hydrophobic interactions between the ligand and the target [20], while high affinity often relies on some crucial interactions such as hydrogen-bonding. So the quantification of both affinity and specificity rather than affinity alone will contribute to the fundamental understanding of molecular recognition.

On the other hand, the correlation between on time of ligand binding to affinity is weak (S2A Fig). The residence time has certain degree of negative correlation with affinity (S2B Fig). This might be due to the increase of the free energy barrier for escaping the binding from the increase of the affinity. The correlation of residence time with respect to affinity is also with spread. But the dispersion is narrower than the correlation between affinity to intrinsic specificity or on time of ligand binding. Therefore, in general, since there is no perfect correlation between affinity and specificity at the thermodynamic or kinetic level, one should consider both of them in exploring the biomolecular recognition.

It should be noted that the interplay between affinity and specificity has also motivated the design of the new score functions of molecular docking [21, 67, 68] by optimizing the corresponding interplays in molecular binding. These further indicate that one needs a comprehensive and multi-dimensional statistical characterizations for molecular recognition.

From these findings, we can provide an integrated quantitative label or indicator for each ligand. So this statistically characterizes each ligand binding to the specific receptor protein with a few characteristic parameters or indicators such as affinity and specificity. When applies to drug screening, this suggests a multi-dimensional screening strategy using both affinity and specificity rather than affinity alone in the traditional drug discovery.

The statistical methodology and approach based on energy landscape theory is quite general, one expects to apply not only to protein-protein/ligand bindings, but also protein-RNA, protein-DNA and RNA-DNA bindings. For more general interactions such as protein-protein interactions and protein nuclei acid interactions, we have performed the investigations on their underlying natures [67, 68]. These studies are on the affinity and specificity at the mean level. We plan to generalize our statistical study here for ligand protein interactions to protein-protein and protein-nuclei acid interactions. For rigid binding, we expect similar statistical behaviors. We can take into account flexible binding at the local level by sampling all the important conformations. However, for flexible binding at the global level, it is still a challenge. Some initial efforts have already been made towards this at the mean level that we can use to explore further the associated statistics [69].

In this paper, to the first order approximation, we have ignored correlations between different states. It is expected that the correlations will influence the tail properties of the statistical distribution of the physical relevant variables quantitatively. It will be interesting to extend the current study to incorporate this effect [70, 71].

## Materials and Methods

### Analytical Models for Binding

Let us now start to work out the functional form of the distribution of the free energy of the ligand-receptor binding complex by exploring the sequence space of different ligands. In order to obtain the free energy, one has to study the thermodynamics of the system. One can do so by starting with an order parameter characterizing the system. A natural choice of the order parameter is the contact variable defined as the contact probability between an atom of the receptor protein and an atom in a small molecule ligand: *σ*_*ij*_ (*σ*_*ij*_ = 1 when *d* < Δ and *σ*_*ij*_ = 0 when *d* > Δ) where Δ is a fixed cutoff distance usually on the order of several angstrom and d is the distance between two residues (or two atoms in the case of small molecule binding with a receptor) which measures how close the two residues are to each other.

The interaction hamiltonian or the energy function of the system in terms the contact variables can be written as

$$\begin{array}{c} H=\sum_{ij}J_{ij}\sigma_{ij} \end{array}$$ (10)

whereas *J*_*ij*_ is the coupling strength between the one atom on a receptor and another one on a ligand. Of course, one can include more complicated form of interactions such as multi-body interactions which mimic the hydrophobic interactions and non-additive properties of the interactions. But this will not influence the general features of the system. We will therefore postpone the discussion on more complicated forms of hamiltonian in the future.

It is worth noticing that this form of the interaction hamiltonian has been widely used in analytical models [70–72], in lattice simulation models [73, 74], off-lattice models [75, 76] of protein folding and protein-structure predictions [23–25].

The coupling strength *J*_*ij*_ between the two atoms can have variety of values. This is because there are n different kinds of atoms, so there are about *n*(*n* − 1)/2 different kinds of interaction strengths at a particular distance between the two atoms. If we variate the distances, the number of interactions will be even more. On the other hand, the purpose of this paper is to study the ensemble of different ligands binding to the receptor. The ensemble of sequences is equivalent to the ensemble of interactions under the ergodicity hypothesis. In other words, changing the sequences of atoms is equivalent to changing the interactions. Therefore by shuffling sequences, one equivalently goes through different interactions. Based on this argument of the equivalence between many possibilities of interaction types due to the multiple types of atoms and ensemble of sequences of ligands, the coupling strengths *J*_*ij*_ can be seen to have a distribution. According to the large number theorem in statistics, this distribution is approximately Gaussian, that is:

$$\begin{array}{c} f\left(J_{ij}\right)∼exp\left[-\frac{{\left(J_{ij}-\bar{J}\right)}^{2}}{2\Delta J^{2}}\right] \end{array}$$ (11)

where $\bar{J}$ is the average coupling strength and Δ*J* ^2^ is the variance. The distribution of the energy of the system can be obtained by calculating < *δ*(*E* − *H*) > where average is over interaction coupling strengths *J*_*ij*_. It is also Gaussian distributed:

$$\begin{array}{c} f\left(E\right)∼exp\left[-\frac{{\left(E-\bar{E}\right)}^{2}}{2\Delta E^{2}}\right] \end{array}$$ (12)

where $\bar{E}$ is the average energy of the system and Δ*E* ^2^ = *N*Δ*J* ^2^ is the variance. N is the total number of contacts. If we ignore the correlations between the different energy states, the result is an independent random energy model [36, 37].

### Simulations

While analytical model can give statistical features, we will further initiate a microscopic atomic detailed level investigation of the significance and implications of ligand binding with COX-2 as a model system. Initially a diversity set of 720 small molecules were selected from the NCI-Diversity database [49] having molecular weights similar to that of the reference compound SC-558, for which the crystal structure of the COX-2 complex is available (PDB code 1CX2) [50, 51]. All conformers of each of the 720 selected molecules were docked with COX-2 using AutoDock [52] to generate a thermodynamic binding energy landscape for each. From this data, the affinity and also intrinsic specificity quantified by ISR for each molecule with COX-2 were calculated as defined above and in Fig 2B.

To study the kinetics of ligand-receptor binding, we need to define an order parameter to describe the binding process on the statistical energy landscape. In this report, we use the RMSD as an order parameter (RMSD represents the root mean square distance relative to the native binding structure) that represents the progress of binding towards native state. For activation process, the order parameter or reaction coordinate RMSD is likely to be locally connected. It is straight-forward to see that the overall kinetic process involves diffusion in order parameter RMSD space. In other words, one can start with a general kinetic master equation, by assuming the local connectivity in RMSD, derive a diffusion equation [26–30, 32]:

$$\begin{array}{c} \frac{\partial }{\partial t}P\left(RMSD,t\right)=\frac{\partial }{\partial RMSD}\left[D\left(RMSD\right)\frac{\partial P(RMSD,t)}{\partial RMSD}+P\frac{\partial (F\left(RMSD\right)/\kappa_{B}T)}{\partial RMSD}\right] \end{array}$$ (13)

where P(RMSD,t) is the probability of the biomolecular binding complex with specific RMSD at time t, D(RMSD) is the diffusion coefficient and F(RMSD) is the free energy of the system at RMSD. The diffusion coefficient is essentially the average time leaving the local minimum energy site. In terms of the order parameter RMSD, the problem becomes one dimensional diffusion. The diffusion equation can be integrated to give the mean first passage time:

$$\begin{array}{c} \bar{\tau }=\int_{RMSD_{i}}^{RMSD_{f}}dRMSD\int_{RMSD_{i}}^{RMSD}dRMSD^{\prime }\frac{exp[\frac{F\left(RMSD\right)-F\left(RMSD^{\prime }\right)}{\kappa_{B}T}]}{D(RMSD)} \end{array}$$ (14)

*RMSD*_*i*_ ∼ 1*A* is where the native binding state is. The boundary conditions for the Fokker-Planck equation are set as a reflecting one at *RMSD*_*i*_, where system is in native state:$[P(RMSD,t)\frac{\partial }{\partial RMSD}F(RMSD)+\frac{\partial }{\partial RMSD}P(RMSD,t)]{|}_{RMSD=RMSD_{i}}=0$, and an absorbing one at *RMSD* = *RMSD*_*f*_, where the system is in the nonnative states: *P*(*RMSD*_*f*_, *t*) = 0. This is for getting the off rate of binding. To get the on rate of binding, we just reverse the order of the reflecting (at non-native) and absorbing boundary condition (at native). The choice of an absorbing boundary condition facilitates the calculation for the first passage time and its distribution.

## Supporting Information

S1 Fig the relationship between the predicted affinity and specificity(ISR) for 720 drugs against the Cox-2. (EPS)

S2 Fig the relationship between the predicted affinity and the predicted kinetic specificity for 720 drugs against the Cox-2. (EPS)

S1 Table Selective (bold) and non-selective nonsteroidal anti-inflammatory drugs(NSAIDs) of COX-2. The predicted affinity (E^*pred*^(uM)) and residence time (${\text{Time}}_{off}^{pred}$) are shown for the drugs. The IC_50_((uM)) and the corresponding affinities (E^*exp*^) for 20 drugs and experimentally determined half life (= 0.693*residence time, ${\text{Time}}_{off}^{exp}$(hr)) for 22 drugs are also listed.
(DOC)

S1 Text The derivation for the free energy distribution. (DOCX)

S2 Text The fitting procedure for the simulation results. (DOC)
