# Supplementary material for: The Universal Statistical Distributions of the Affinity, Equilibrium Constants, Kinetics and Specificity in Biomolecular Recognition
Source: PLoS Comput Biol. 2015 Apr 17;11(4):e1004212. doi: 10.1371/journal.pcbi.1004212 (PMC4401658; doi:10.1371/journal.pcbi.1004212)
Supplement: S1 Table — The predicted affinity (Epred(uM)) and residence time (Timeoffpred) are shown for the drugs. The IC50((uM)) and the corresponding affinities (Eexp) for 20 drugs and experimentally determined half life (= 0.693*residence time, Timeoffexp(hr)) for 22 drugs are also listed. (DOC) [file pcbi.1004212.s003.doc]

Table S1. selective (bold) and non-selective nonsteroidal anti-inflammatory drugs(NSAIDs) of COX-2. The predicted affinity (E^pred^(uM)) and residence time (Time_off_^pred^) are shown for the drugs. The IC_50_ ((uM)) and the corresponding affinities (E^exp^) for 20 drugs and experimentally determined half life (=0.693*residence time, Time_off_^exp^(hr)) for 22 drugs are also listed.

| **Drugs** | **IC_50_** | **E^exp^** | **E^pred^** | **Time_off_^pred^** | **Time_off_^exp^** |
| --- | --- | --- | --- | --- | --- |
| **ns-398** | 0.47 | -8.686 | -8.16 | 3798.10 | N/A |
| **l-745337** | 9.67 | -6.884 | -8.19 | 4436.85 | N/A |
| **celecoxib** | 0.87 | -8.319 | -9.79 | 14570.29 | 11.2 |
| **rofecoxib** | 0.53 | -8.615 | -8.80 | 6436.09 | 17.0 |
| **dup-697** | 0.06 | -9.913 | -9.65 | 22077.36 | 292.0 |
| **jte-522** | 0.085 | -9.706 | -9.85 | 10240.28 | N/A |
| **valdecoxib** | 0.87 | -8.319 | -9.33 | 7266.53 | 9.5 |
| **etoricoxib** | 1.10 | -8.179 | -9.35 | 14095.39 | 22.0 |
| **meloxicam** | 0.70 | -8.449 | -8.75 | 5228.94 | 17.5 |
| **etodolac** | 3.70 | -7.456 | -7.47 | 2317.83 | 7.3 |
| **l-776967** | 0.03 | -10.327 | -8.47 | 5892.80 | N/A |
| **flosulide** | 0.75 | -8.408 | -7.88 | 3837.76 | N/A |
| **sulindac-sulfide** | 10.43 | -6.838 | -7.24 | 1637.74 | 16.4 |
| **tolmetin** | 7.09 | -7.069 | -7.79 | 2568 | 7.0 |
| **ketoprofen** | 1.08 | -8.19 | -7.94 | 2730.64 | 2.6 |
| **ketorolac** | 0.86 | -8.326 | -7.54 | 1558.48 | 3.8 |
| **Ibuprofen** | 24.3 | -6.334 | -6.32 | 1431.87 | 3.0 |
| **flurbiprofen** | 6.42 | -7.128 | -7.36 | 1610.21 | 5.2 |
| **tenoxicam** | 14.22 | -6.653 | -7.06 | 3173.08 | N/A |
| **piroxicam** | 9.00 | -6.92 | -8.75 | 3068 | 5.0 |
| **cis-stilbenes** | N/A | N/A | -7.74 | 3289.28 | 21.5 |
| **carprofen** | N/A | N/A | -8.19 | 2880.37 | 7.2 |
| **fenoprofen** | N/A | N/A | -7.50 | 2846.01 | 3.0 |
| **Indoprofen** | N/A | N/A | -8.50 | 4334.26 | 2.3 |
| **loxoprofen** | N/A | N/A | -7.56 | 2083.24 | 1.2 |
| **meclofenamic-acid** | N/A | N/A | -7.51 | 2379.49 | 3.1 |
| **oxaprozin** | N/A | N/A | -8.09 | 4004.59 | 54.9 |
| **Indomethacin** | N/A | N/A | -8.60 | 4808.33 | 4.5 |
