# Supplementary material for: The Universal Statistical Distributions of the Affinity, Equilibrium Constants, Kinetics and Specificity in Biomolecular Recognition
Source: PLoS Comput Biol. 2015 Apr 17;11(4):e1004212. doi: 10.1371/journal.pcbi.1004212 (PMC4401658; doi:10.1371/journal.pcbi.1004212)
Supplement: S1 Text — (DOCX) [file pcbi.1004212.s004.docx]

The free energy distribution

The Hamiltonian or energy function for the interactions between ligands and receptors can be described by the collections of contact interactions between the atom pairs $E=-\sum_{ij} J_{ij}\sigma_{ij}$,where $\sigma_{ij}$ is the contact variable between atoms with certain distance cutoff and the $J_{ij}$ is the coupling strength for specific contact pair. Since there are many different types of atoms and also many different cutoff distances for the interactions, different $J_{ij}$s have different values. This forms a distribution for the coupling J. Since the number of different $J_{ij}$ couplings are large, the statistical distribution should have a Gaussian form from the large number theorem. Then we can prove that this is equivalent to a random energy model with the interaction energy follows a Gaussian distribution[19]. This reflects the complexity of the underlying interactions in contrast to the conventional models for simple systems where the coupling strengths are fixed and not distributed.

The resulting random energy model can be defined as follows:

(1). The system has M^N^ energy levels E_i_ where M is the number of the configurations for unit while N is the number of units.

(2) These energy levels are distributed according to the probability distribution $P(E)=(N\pi J^{2})^{-1/2}\exp(-E^{2}/NJ^{2})$. J is the coupling strength between contacts.

(3). The energy leves E_i_ are independent random variables.

From the random energy model, we can explore the statistical properties of associated free energy ( 37).

The system composes of M^N^ energy levels E_i_. The corresponding partition function Z is then given by $Z(\{E_{i}\})=\sum_{i=1}^{2^{N}} \exp\left\{-\frac{E_{i}}{T}\right\}$. Once the partition function Z is known, the free energy is known.

Notice that since the energy is distributed due to the statistical properties of the coupling strengths, we expect the corresponding partition function is distributed. Therefore, we expect the corresponding free energy is also distributed. We can calculate the average free energy by the formula:

Furthermore, the free energy can be obtained using the formula:$F=-\kappa_{B}T\ln Z$(here,k_B_ =1). Based on the previous studies[36], we can reach the form of the probability distribution P(Z) of Z from the distribution of the energy.

After some algebra, the distribution of partition function reads: $P(Z)dZ=g(\frac{Z}{Z_{0}})d(\frac{Z}{Z_{0}})$for $\frac{Z}{Z_{0}}$finite;

The distribution of partition function reads:

$p(Z)dZ=2^{N}\sqrt{\frac{T^{2}}{NJ^{2}\pi}}\exp(-\frac{T^{2}}{NJ^{2}}\ln Z^{2})d\ln Z$ for $\frac{Z}{Z_{0}}$ exponentially large in N.

The moments <Z^v^> of the partition function is: $<Z^{v}>=\int...\int\mathop{\Pi}\limits_{i=1}^{2^{N}} P(E_{i})dE_{i}$ and the function g(x) is $\int x^{v}g(x)dx=\left\{\begin{matrix} \frac{\Gamma(1-vT_{c}/T)}{\Gamma(1-v)}(v<T/Tc) \\ \infty(v>T/Tc) \end{matrix}\right.$.

When v>T/T_c_ , the formula $p(Z)dZ=2^{N}\sqrt{\frac{T^{2}}{NJ^{2}\pi}}\exp(-\frac{T^{2}}{NJ^{2}}\ln Z^{2})d\ln Z$ dominates the integral $\int Z^{v}P(Z)dZ$ and the moments $<Z^{v}>\sim2^{N}\exp[Nv^{2}J^{2}/4T^{2}]$ for T>T_c_ and v>(T/ T_c_)^2^ or T< T_c_ and v>T/ T_c_. can be recovered. Therefore, by the formula $p(Z)dZ=2^{N}\sqrt{\frac{T^{2}}{NJ^{2}\pi}}\exp(-\frac{T^{2}}{NJ^{2}}\ln Z^{2})d\ln Z$ and F=-TlnZ(k_b_=1), we can easily obtain the gaussian distribution: P(F) ~$\exp(-\frac{F^{2}}{NJ^{2}})$ as shown in equation 1 in the main text. In the paper, we use the f(F) to represent the free energy distribution. Notice that the g(x) mentioned above has an asymptotic form $g(x)\sim x^{-T/T_{c}-1}(-\frac{1}{\Gamma(-T/T_{c})})$ for $x\to\infty$. Based on the formula$P(Z)dZ=g(\frac{Z}{Z_{0}})d(\frac{Z}{Z_{0}})$,one can also recover the moments $<Z^{v}>=\int Z^{v}g(\frac{Z}{Z_{0}})d(\frac{Z}{Z_{0}})$ for T<T_c_ and v<T/T_c_ . Therefore, we can obtain the distribution P(Z) of Z at the tail similar to g(x) above. Since partition function Z is power law distributed asymptotically at the tail, the associated free energy is therefore exponentially distributed at the tail （$x\to\infty$）as shown in equation 2 in the main text. In other words, for the distribution of free energy, we can obtain two distinct regions (low and high free energy) of the corresponding physical variable.The exponential distribution of free energy can also be obtained by the formula (3)[37]. The authors had succeeded in proving the formula (3) by computing the inclusive distributions of probabilities f^(k)^.
