# Supplementary material for: The Universal Statistical Distributions of the Affinity, Equilibrium Constants, Kinetics and Specificity in Biomolecular Recognition
Source: PLoS Comput Biol. 2015 Apr 17;11(4):e1004212. doi: 10.1371/journal.pcbi.1004212 (PMC4401658; doi:10.1371/journal.pcbi.1004212)
Supplement: S2 Text — (DOC) [file pcbi.1004212.s005.doc]

The fittings for the simulation results

The aim of nonlinear fitting is to get the parameter values which best describe the simulation results. The standard way of finding the best fit is to choose the parameters that would minimize the deviations of the theoretical curve(s) from the experimental points. We call the method as chi-square minimization.

$$X^{2}=\sum_{i=1}^{n} \left[\frac{Y_{i}-f(x_{i}^{'},\theta^{\wedge} )}{\sigma_{i}}\right]^{2}$$

Where $x_{i}^{'}$is the row vector for the ith (i = 1, 2, ... , n) observation. Where X=(x_1_, x_2_,… x_k_)' is the independent variables and θ= (θ_1_,θ_2_, …θ_k_)' is the parameters. To estimate the $\theta^{\wedge}$ value with the least square method, we need to solve the normal equations which are set to be zero for the partial derivatives of $X^{2}$ with respect to each $\theta^{\wedge} _{p}$.

In the study, we can't find a suitable fitting function in the built-in function library of the Origin package. Then we have defined a new fitting function for use in nonlinear fitting based on our analytical model, defined as follows:

$y=y0+A1e^{-0.5k1(\left|x-xc1\right|+(xc1-x))}+\frac{A2}{w\sqrt{\frac{\pi}{2}}}e^{-\frac{2(x-xc2)^{2}}{w^{2}}}+A3e^{-0.5k2(\left|x-xc3\right|+(x-xc3))}$We employ an iterative strategy to estimate the parameter values due to the difficulty of getting the explicit solutions to the equations. This process starts with some initial values, with each iteration, a $X^{2}$ value is computed and then the parameter values are adjusted so as to reduce the $X^{2}$. When the $X^{2}$ values computed in two successive iterations are small enough(compared with the tolerance, herein, the tolerance is set to 1E-15, the maximum number of iterations is set to 50000), the fitting procedure has converged. We have also chosen the option ”reduced chi-square” during the fitting procedure. The Levenberg-Marquardt (L-M) algorithm is used to adjust the fitting parameter values in the above iterative procedure in the current study. This algorithm combining the Gauss-Newton method and the steepest descent method works for most cases. In order to get a good value for parameter initialization, the function peak_pos is used to estimate the peak's XY coordinate, peak's width, area.etc for the Gauss distribution. There are two methods to calculate the confidence intervals for parameters: Asymptotic-Symmetry method and Model-Comparison method in the Origin package. We applied the former to obatin asymptotic, symmetrical confidence intervals. All Curve-fittings are carried out with software Origin ([www.originlab.com](http://www.originlab.com/)).

We have listed the fitting reports for global fit including the Parameters(See Figure4-8), Statistics and ANOVA tables for each physical variable.

The ANOVA table of nonlinear fitting for these four physical variables:

Time_on_:

|  |  | DF | Sum of Squares | Mean Square | F Value | Prob>F |
| --- | --- | --- | --- | --- | --- | --- |
| Count | Regression | 10 | 46247.74645 | 4624.77464 | 72.00855 | 6.6228E-8 |
|  | Residual | 10 | 642.25355 | 64.22536 |  |  |
|  | Uncorrected Total | 20 | 46890 |  |  |  |
|  | Corrected Total | 19 | 25241.8 |  |  |  |

Time_off_:

|  |  | DF | Sum of Squares | Mean Square | F Value | Prob>F |
| --- | --- | --- | --- | --- | --- | --- |
| Count | Regression | 10 | 47221.86487 | 4722.18649 | 92.20587 | 1.9752E-8 |
|  | Residual | 10 | 512.13513 | 51.21351 |  |  |
|  | Uncorrected Total | 20 | 47734 |  |  |  |
|  | Corrected Total | 19 | 26085.8 |  |  |  |

Specificity(ISR):

|  |  | DF | Sum of Squares | Mean Square | F Value | Prob>F |
| --- | --- | --- | --- | --- | --- | --- |
| Count | Regression | 10 | 42785.81892 | 4278.58189 | 226.1633 | 2.3530E-10 |
|  | Residual | 10 | 189.18108 | 18.91811 |  |  |
|  | Uncorrected Total | 20 | 42975 |  |  |  |
|  | Corrected Total | 19 | 16548.55 |  |  |  |

Equilibrium constant:

|  |  | DF | Sum of Squares | Mean Square | F Value | Prob>F |
| --- | --- | --- | --- | --- | --- | --- |
| Count | Regression | 10 | 54950.94654 | 5495.09465 | 37.32945 | 1.5834E-6 |
|  | Residual | 10 | 1472.05346 | 147.20535 |  |  |
|  | Uncorrected Total | 20 | 56423 |  |  |  |
|  | Corrected Total | 19 | 29996.55 |  |  |  |

Affinity:

|  |  | DF | Sum of Squares | Mean Square | F Value | Prob>F |
| --- | --- | --- | --- | --- | --- | --- |
| Count | Regression | 10 | 57587.75475 | 5758.77547 | 52.38845 | 3.1067E-7 |
|  | Residual | 10 | 1099.24525 | 109.92453 |  |  |
|  | Uncorrected Total | 20 | 58687 |  |  |  |
|  | Corrected Total | 19 | 32260.55 |  |  |  |
